# Identification and Characterization of a New *Serratia proteamaculans* Strain That Naturally Produces Significant Amount of Extracellular Laccase

**DOI:** 10.3389/fmicb.2022.878360

**Published:** 2022-07-18

**Authors:** Nadia Sufdar Ali, Fang Huang, Wensheng Qin, Trent Chunzhong Yang

**Affiliations:** ^1^Department of Biology, Lakehead University, Thunder Bay, ON, Canada; ^2^Aquatic and Crop Resource Development Research Centre, National Research Council, Ottawa, ON, Canada

**Keywords:** bacterial laccase, lignin degradation, lignocellulose, screening, *Serratia proteamaculans*, submerged culture

## Abstract

Natural biodegradation processes hold promises for the conversion of agro-industrial lignocellulosic biomaterials into biofuels and fine chemicals through lignin-degrading enzymes. The high cost and low stability of these enzymes remain a significant challenge to economic lignocellulosic biomass conversion. Wood-degrading microorganisms are a great source for novel enzyme discoveries. In this study, the decomposed wood samples were screened, and a promising γ-proteobacterial strain that naturally secreted a significant amount of laccase enzyme was isolated and identified as *Serratia proteamaculans* AORB19 based on its phenotypic and genotypic characteristics. The laccase activities in culture medium of strain AORB19 were confirmed both qualitatively and quantitatively. Significant cultural parameters for laccase production under submerged conditions were identified following a one-factor-at-a-time (OFAT) methodology: temperature 30°C, pH 9, yeast extract (2 g/l), Li^+^, Cu^2+^, Ca^2+^, and Mn^2+^ (0.5 mM), and acetone (5%). Under the selected conditions, a 6-fold increase (73.3 U/L) in laccase production was achieved when compared with the initial culturing conditions (12.18 U/L). Furthermore, laccase production was enhanced under alkaline and mesophilic growth conditions in the presence of metal ions and organic solvents. The results of the study suggest the promising potential of the identified strain and its enzymes in the valorization of lignocellulosic wastes. Further optimization of culturing conditions to enhance the AORB19 strain laccase secretion, identification and characterization of the purified enzyme, and heterologous expression of the specific enzyme may lead to practical industrial and environmental applications.

## Introduction

Laccases (benzenediol: oxygen oxidoreductases, EC 1.10.3.2) are copper-containing oxidoreductases that can oxidize a wide range of phenolic and nonphenolic substrates, including ortho, meta, and para diphenols, specifically lignin, a lignocellulose component, and is employed as an attractive tool for the pre-treatment of biomass and its valorization. They are versatile oxidative biocatalysts that contain copper atoms in their active site and oxidize diverse substrates using only molecular oxygen as the known co-substrate instead of hydrogen peroxide, as in peroxidases (Agrawal et al., 2018; Agrawal and Verma, 2020). As green biocatalysts, laccases have been exploited for a potential application in broad biotechnological areas, viz., environmental remediation processes, biosensor design, synthesis of fine chemicals, food, cosmetic, and pharmaceutical industries, and synthetic dye decolorization capacity to detoxify a range of noxious and recalcitrant environmental pollutants (Yang et al., 2017; Becker and Wittmann, 2019; Zerva et al., 2019). However, most currently known laccases are difficult to overproduce in heterologous hosts (Kim et al., 2010). Abundant and inexpensive laccases are needed if the microbiological treatments are to compete with chemical treatments in industries to which they appeal.

Identification and engineering for more efficient and tolerant laccases for industrial applications are an ongoing effort to date. Laccase genes dwell in numerous biologically important taxa including plants, insects, lichen, bacteria, and fungi, with the Basidiomycetes class of fungi being the most important source (Hoegger et al., 2006; Arregui et al., 2019). It has been reported that microbial laccases are primarily involved in wood decay, lignin decomposition, and detoxification and linked to resistance to different environmental stresses (Arregui et al., 2019; Janusz et al., 2020). Fungal laccases have been studied in a variety of biotechnological applications due to their high redox potential (Abdel-Hamid et al., 2013; Upadhyay et al., 2016). However, their application is usually hampered due to the long fermentation periods, acidic pH optima, intolerance to extreme conditions, and difficulty in overproducing in heterologous hosts (Baldrian, 2006; Kim et al., 2010; Du et al., 2015). Meanwhile, small laccases, both three- and two-domain laccases, from bacterial sources have gained attention recently, due to their exceptional attributes such as ability to withstand wide temperature and pH ranges, ease in genetic manipulation, and tremendous stability even when inhibitory agents are present (Mukhopadhyay et al., 2013; Chauhan et al., 2017; Arregui et al., 2019; Gianolini et al., 2020; Sharma and Leung, 2021). In addition, a short generation time of bacteria makes it easier to scale up laccase production processes on a commercial scale (Brugnari et al., 2021; Akram et al., 2022). The major bacterial genera that have been reported to produce laccase-like multicopper oxidases include Streptomyces, Bacillus, Meiothermus, Gramella, Geobacillus, Aquisalibacillus, Lysinibacillus, Azospirillum, Rhodococcus, Ochrobactrum, Amycolatopsis, Pseudomonas, Stenotrophomonas, Iodidimonas, Alteromonas, and Nitrosomonas (Chauhan et al., 2017; Granja-Travez et al., 2018; Arregui et al., 2019; Jeon and Park, 2020; Yang et al., 2021).

Bacterial species under the genus Serratia, belonging to the family Enterobacteriaceae, has been identified for its numerous applications in biodegradation (Majumdar et al., 2020; An et al., 2021; Dabrowska et al., 2021). *Serratia proteamaculans*, a gram-negative, non-pigmented strain under this genus, was first reported to cause a leaf spot disease and is the only identified phytopathogen under the genus Serratia (Paine and Standsfield, 1919; Mahlen, 2011). It is widely distributed in nature and is frequently isolated from the gut microbiota of insects, including spiders and bark beetles (Bersanetti et al., 2005; Mikhailova et al., 2014). *Serratia proteamaculans* has been recently recognized for its capability to produce bio-degradative enzymes (Mehmood et al., 2009; Madhuprakash et al., 2015; Cano-Ramírez et al., 2016) and possess remarkable antagonistic traits against plant pathogens (Wang et al., 2014).

Even though its ability to synthesize extracellular enzymes such as chitinase, endoglucanase, and protease has been reported (Mikhailova et al., 2014; Madhuprakash et al., 2015; Cano-Ramírez et al., 2016), no research regarding its growth and extracellular laccase production has been reported so far. During an investigation of the cultivable microbial community from decomposed wood on the Ottawa River bank, a large number of bacterial and fungal strains were isolated. Among them, a new strain of *Serratia proteamaculans*, AORB19, has been isolated and characterized that showed significant laccase activities during the screening process. This study characterized the identified strain AORB19 and investigated its laccase production potential and the important culture parameters for enhanced laccase production under submerged culture conditions.

## Materials and Methods

### Chemicals

The following chemicals were used: 2-2′-azino-bis-[3-ethyl benzthiazoline-6-sulfonic acid] (ABTS), 2,6-dimethoxy phenol (DMP), syringaldazine (SGZ), kraft lignin, guaiacol, and Czapek–Dox (CDA) broth. All of the compounds were analytical grade and purchased from Sigma-Aldrich in Canada.

### Organism Screening and Growth Conditions

The decomposed wood samples were collected near the Ottawa River bank (geographical coordinates: 45°26′59.7″N, 75°41′23.3″W). The microorganisms were isolated from the decomposed wood samples by an enrichment culture method using Czapek–Dox broth (L-CDB) with 0.1% lignin alkali (kraft 98%, Sigma-Aldrich, Canada, MW>28,000). The best strain was selected on the basis of copious and stable laccase activity for a prolonged time period in the culture medium. The pure culture of the organism was maintained in glycerol stocks, and fresh cultures from overnight incubation were used in all experiments. The bacterium isolated from the wood samples was phenotypically characterized based on the physiological, morphological, and biochemical tests. Besides, the bacterium was also further characterized and determined based on 16S rDNA sequencing.

### Phenotypic Characterization of the Bacterial Strain

A bacterial isolate was phenotypically analyzed for presumptive identification and categorized based on cell morphology, gram's reaction, colony morphology, and growth at various pH and temperatures.

#### Biochemical Characterization of the Bacterial Strain

The bacterial strain was identified using a Microscan conventional biochemical detection system for a gram-negative bacilli (NID2) panel (Beckman Coulter, Brea, CA, USA) with dehydrated substrates. pH variations, substrate utilization, and growth in the presence of antimicrobials were used to identify the bacterial strain. The panels were inoculated with bacterial culture and incubated following the manufacturer's instructions (Ombelet et al., 2021). The bacterial strain with a probability score was identified based on the Beckman Coulter program.

### Molecular Identification of Bacteria

DNA preparation, PCR amplification, and 16S rRNA gene sequencing analysis.

The genomic DNA was extracted using a bacterial DNA isolation kit (Bio Basic, Canada). The universal eubacterial primers 27F (5′-AGAGTTTGATCMTGGCTCAG-3′) and 1492R (5′-CGGTTACCTTGTTACGACTT-3′; Narde et al., 2004) were used to amplify the 16S rRNA gene. The reaction mixture contained in a final reaction volume of 50 μL with 5 μL of DNA template, 200 μM of dNTPs, 1 × Phusion buffer, 0.5 μM of primer, and one unit of Phusion DNA polymerase (Thermo Fisher Scientific, USA). The PCRs were carried out on a Bio-Rad T100 Thermal Cycler. Thirty-five cycles were performed to amplify the 16S rRNA gene following an initial denaturation at 95°C for 2 min, a subsequent denaturation at 95°C for 30 s followed by annealing at 52°C for 30 s, an extension at 72°C for 2 m, and a final extension at 72°C for 15 m. The PCR products were then resolved on a 1% agarose gel and purified using a Qiagen quick gel extraction kit. The purified PCR product was sequenced by Eurofins genomics in Canada for 16S rRNA gene sequencing analysis.

Chromas Lite 2.0 software (https://technelysium.com.au/wp/chromas) was used to assess the quality of the sequences acquired. After aligning the forward and reverse sequences with BioEdit software, a conserved sequence (1404bp) was created. BLAST analyses of the conserved sequence were performed (https://blast.ncbi.nlm.nih.gov/Blast.cgi) to identify the sequences that show a maximum identity to those in GenBank. Multiple sequence alignment analysis of 18 type strains that showed the highest identity and similarity to the bacterial isolate's sequence was performed using the Clustal W program. MEGA X program was used to determine the evolutionary relationships between closely related sequences using multiple alignment files. The evolutionary history was inferred using the neighbor-joining (NJ) criterion, and a bootstrap analysis was performed using 1,000 pseudo-replicates.

### Laccase Activity Measurement

#### Qualitative Laccase Assay

2-2'-Azino-bis-[3-ethyl benzthiazoline-6-sulfonic acid] and guaiacol plate assay methods were used to perform a qualitative laccase activity assay (Senthivelan et al., 2019). ABTS and guaiacol were used as substrates to screen the isolated bacterium for laccase enzyme. Individually, the bacteria were inoculated on CDA agar plates with 3 mM ABTS and 4 mM guaiacol and incubated at 27°C. The culture plates were visually observed for color change and formations in the media. Laccase production is indicated by a green or brown halo around the bacterial colony on ABTS and guaiacol agar, respectively.

#### Quantitative Laccase Assay

A 24-h-old freshly grown bacterial colony (O.D.600 nm ± 0.05) was inoculated aseptically onto 50 mlCzapek–Dox broth (pH 7) with 0.1% lignin alkali (kraft 98%, Sigma-Aldrich, Canada, MW>28,000) at 27°C with 125 rpm shaking. The broth culture was centrifuged for 5 min at 13,000 rpm at 4°C after the incubation period, and the supernatant was used as the enzyme source.

A laccase activity was determined using 2,6-dimethoxyphenol (DMP) in sodium acetate buffer (0.1 M) with pH 5, and quantified spectrophotometrically (SpectraMax M5, Molecular Devices, San Jose, CA, USA) at 469 nm ε469 = 14,800 M^−1^ cm^−1^ (Agrawal and Verma, 2019). A total reaction volume of 210 μL was used for the standard assay, which included 160μL 2,6-DMP (10 mM) in sodium acetate buffer (0.1 M, pH 5) and 50 μL crude enzyme. One unit of laccase activity (U) was defined as the amount of the enzyme required to oxidize 1 μmol of 2,6-DMP per minute. A control was run in parallel by replacing the enzyme with buffer under standard conditions. With minor modifications, the laccase activity was determined based on the formula described by Leonowicz and Grzywnowicz (1981):


$$\begin{array}{l} \left(\text{U}/\text{L}\right)=\left(\Delta \text{Abs}\times \text{V}\times 106\right)/\;\left(\varepsilon \times \text{Ve}\times \text{d}\times \Delta \text{t}\right) \end{array}$$

where ΔAbs is the difference in absorbance values; ε is the extinction coefficient; V is the total volume of the sample (mL); Ve is the volume of the enzyme (mL); Δt is the time (min); and d is the path length of microplate.

In addition, the laccase activity was also determined using the following substrates: (a) ABTS (ε_420_ = 36,000 M^−1^cm^−1^; Johannes and Majcherczyk, 2000) in 100 mM sodium acetate buffer with pH 5.0 with 5 mM final concentration of the substrate; (b) syringaldazine (ε _530_ = 65,000 M^−1^cm^−1^; Holm et al., 1998) in 100 mM phosphate buffer with pH 6.0 with 0.22 mM final concentration of the substrate; and (c) L-DOPA (ε _530_ = 3,600 M^−1^cm^−1^; Jara et al., 1988) in 10 mM sodium phosphate buffer with pH 6.8 with 7.6 mM final concentration of the substrate.

### Optimal Culture Conditions for Laccase Production

To investigate the optimal conditions for laccase production, a one-factor-at-a-time (OFAT) strategy was employed on a *Serratia proteamaculans* AORB19 strain. The growth conditions of the bacterial strain were evaluated to identify the best parameters for the maximum laccase production. The tested variables include: physicochemical conditions (temperature and pH), nutritional conditions (carbon sources and nitrogen sources), the addition of organic solvents (acetone, chloroform, and formaldehyde), and metal ions (Mn^2+^, Cu^2+^, Ca^2+^, and Li^+^). In addition, the OD values of liquid culture were measured and tabulated to compare the bacterial cell growth and laccase production. An optical density of bacterial growth was determined spectrophotometrically at λ = 600 nm. The results were analyzed and illustrated graphically by GraphPad Prism software, version 9.0.0.

### Confirmation of *Serratia proteamaculans* Strain AORB19 Laccase Activities

#### Crude Enzyme Preparation

The supernatant was collected by centrifugation at 13,000 rpm for 5 min after the bacterial culture was induced with 0.1 mM ABTS. The supernatant was filtered using a Millex syringe filter unit with a 0.22-m pore size (Millipore Sigma, USA) to remove the intense brownish color of the lignin degradation products from kraft lignin present in the culture medium. The filtrate was concentrated using a centrifugal concentrator (Vacufuge Plus, Canada) under vacuum conditions.

#### SDS-PAGE and Zymogram Analysis

For sodium dodecyl sulfate–polyacrylamide gel electrophoresis (SDS–PAGE), a 12% polyacrylamide slab gel was employed (Laemmli, 1970). Native gel electrophoresis was performed using Tris–glycine as running buffer (pH 8.3), native loading dye (Bio-Rad, USA) under non-reducing and non-denaturing conditions on 10% resolving gel omitting SDS and a heating step to maintain the native state of protein. After that, the samples were electrophoresed at 50 V on the stacking gel (6%) and 100 V on the resolving gel. After electrophoresis, the gel was immersed for 30 min at room temperature in 100 mM sodium acetate buffer (pH 4), which was then submerged in 2 mM ABTS in the same buffer. Laccase activity bands were visualized and confirmed by the appearance of green-colored bands on the gel due to the oxidation of ABTS.

### Statistical Analysis

All experiments were carried out in triplicates (*n* = 3), and the results were expressed as mean±standard error in GraphPad Prism software. Two-way ANOVA with Dunnett's multiple tests was carried out for *post-hoc* comparisons using GraphPad Prism 9.0.0. The Dunnett's range test values were significant (*P* < 0.0001) and non-significant (*P* > 0.05).

## Results

### Phenotypic and Biochemical Characterization of Laccase-Producing Bacterial Strain

The bacterial strain developed whitish, mucoid opaque, convex colonies on Czapek–Dox agar after 24-h incubation at 27°C. The microscopic morphology revealed gram-negative bacilli. The bacterium was facultative anaerobic in nature and able to thrive at temperatures ranging from 20°C to 37°C (optimum 30°C) and pH levels ranging from 6 to 11 (optimum 7–9 pH). The strain was able to utilize five out of nine sugars in carbohydrate fermentation experiments. Furthermore, the strain was positive for lysine, ornithine, galactosidase, citrate, and Voges–Proskauer tests. Negative results were observed with arginine, tryptophan deaminase, indole, malonate, acetamide, tartarate, H_2_S, and esculin hydrolysis. The bacterial strain was found to be resistant to cephalothin (8 g/mL), among all other antibiotics used in the gram-negative ID type 2 (NID2) panel (in Supplementary Table 1).

Following the phenotypical characterization, the bacterial strain was subjected to molecular identification using 16S rRNA gene sequencing. The size of PCR-amplified 16S rRNA gene fragment was approximately 1,500 bp and was indicated by an intact band (Supplementary Figure 1). The purified PCR product was sequenced and showed 99% homology to the 16S rRNA sequence of *Serratia proteamaculans* 336X. A total of 18 deposited 16S rRNA sequences with the highest identities were selected and aligned with the sequenced 16S rRNA. The phylogenetic tree confirmed that the isolated bacterial strain belongs to *Serratia proteamaculans* and was named *Serratia proteamaculans* AORB19 (Supplementary Figure 2).

### Confirmation of *Serratia proteamaculans* AORB19 Laccase Activity

#### Qualitative Laccase Assay

**Figure 1:**
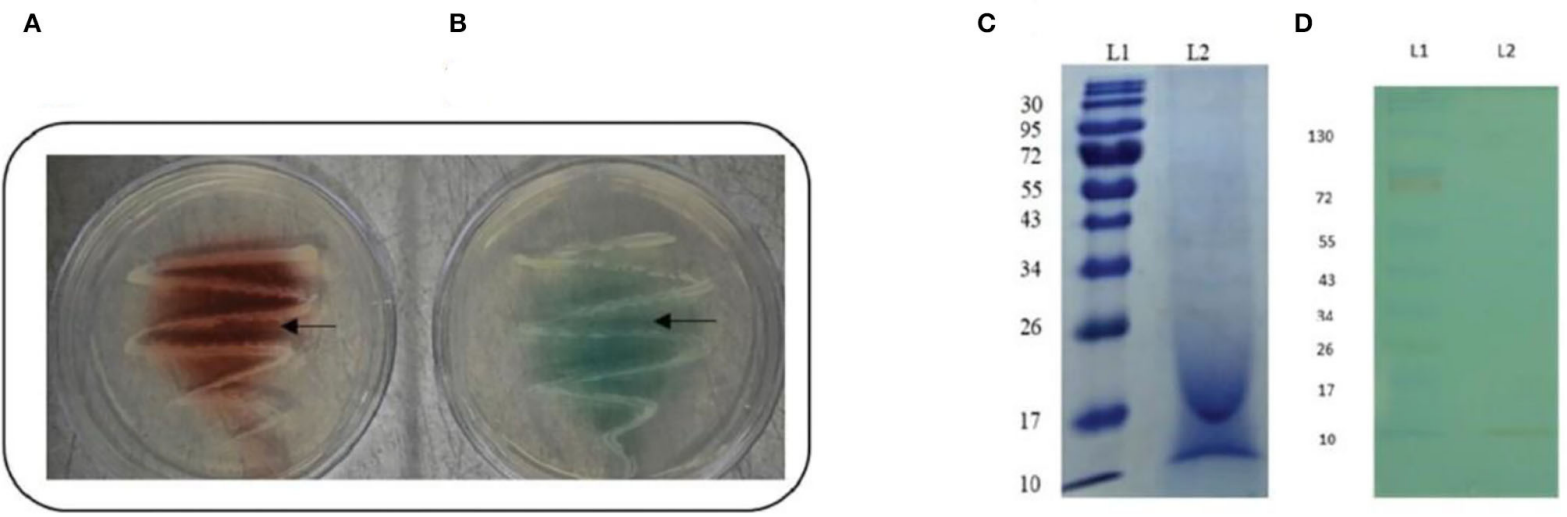
Confirmation of laccase production by strain AORB19. **(A)** Guaiacol plate assay showing reddish brown oxidation zone around bacterial colony; and **(B)** ABTS plate assay showing green oxidation zone around bacterial colony. **(C)** SDS-PAGE analyses of culture supernatant of strain AORB19. L1: Molecular mass marker, L2: Culture supernatant; **(D)** NATIVE - PAGE analyses of culture supernatant of strain AORB19. L1: Molecular mass marker, L2: Laccase activity staining of culture supernatant with ABTS as substrate (green band).

After 48 h of incubation, the isolate's ability to produce laccase enzyme was qualitatively assessed by the formation of a reddish brown-colored zone around colonies on guaiacol agar plates and a green-colored zone around colonies on ABTS agar plates (Figures 1A,B).

#### Quantitative Laccase Assay

The strain AORB19 was incubated on L-CDB medium for 24 h, and the supernatant was used as the crude enzyme to analyze the laccase activity. The laccase activity was measured spectrophotometrically using phenolic substrate 2,6-DMP as a substrate, and its oxidation in the presence of laccase enzyme results in the formation of a stable dimeric form, 3,3P,5,5P-tetra-methoxy-diphenyl-quinone with the appearance of a bright orange color (Solano et al., 2001). The laccase activity was also determined by measuring the rate at which syringaldazine was oxidized to generate tetra-methoxy-azo-bis-methylene quinone, which developed a pink to purple tint (Holm et al., 1998). Furthermore, the oxidation of ABTS to the cation radical ABTS.+ was catalyzed by laccase, as demonstrated by the green–blue color (Johannes and Majcherczyk, 2000). Three distinct substrates, namely, DMP, SGZ, and ABTS, each leading to specific products observable at 469, 530, and 420 nm, respectively, were observed yielding a laccase activity of 0.0384, 0.0062, and 0.0088 μmol/mg/min, respectively.

The culture supernatant of *Serratia proteamaculans* AORB19 was further analyzed for its ability to oxidize ABTS, syringaldazine, and tyrosine spectrophotometrically. Both syringaldazine and ABTS were oxidized, but no positive reaction for tyrosine oxidation was detected (data not shown). The most common substrates used for laccase are 2,6-DMP and guaiacol that can also be oxidized by other enzymes. Enzymes exhibiting the oxidation of ABTS and SGZ, but not tyrosine, are regarded as laccase specifically (Sondhi and Saini, 2019). Our assay results revealed that the bacterial isolate could oxidize ABTS and SGZ, but not tyrosine, indicating that strain AORB19 mostly secreted laccase in L-CDB medium. As L-CDB medium contains lignin as the sole carbon source, it is commendable to suggest that strain AORB19 and its secreted laccases may be applicable for lignin degradation and valorization.

#### In-gel Assay of Strain AORB19 Laccase Activity

The cell culture supernatant of strain AORB19 was analyzed by SDS–PAGE (Figure 1C), and multiple bands were observed, suggesting that a mixture of proteins was secreted in the collected samples. As our assay detects the total laccase activities, it is not sure whether the detected activities were from different enzymes or single enzyme. The supernatant was also analyzed by native PAGE followed by in-gel enzyme activity assay using ABTS as a substrate (Figure 1D). The green band toward the lower molecular end of the gel indicated that our detected laccase activities may be due to a single enzyme.

### Time-Course Study

**Figure 2:**
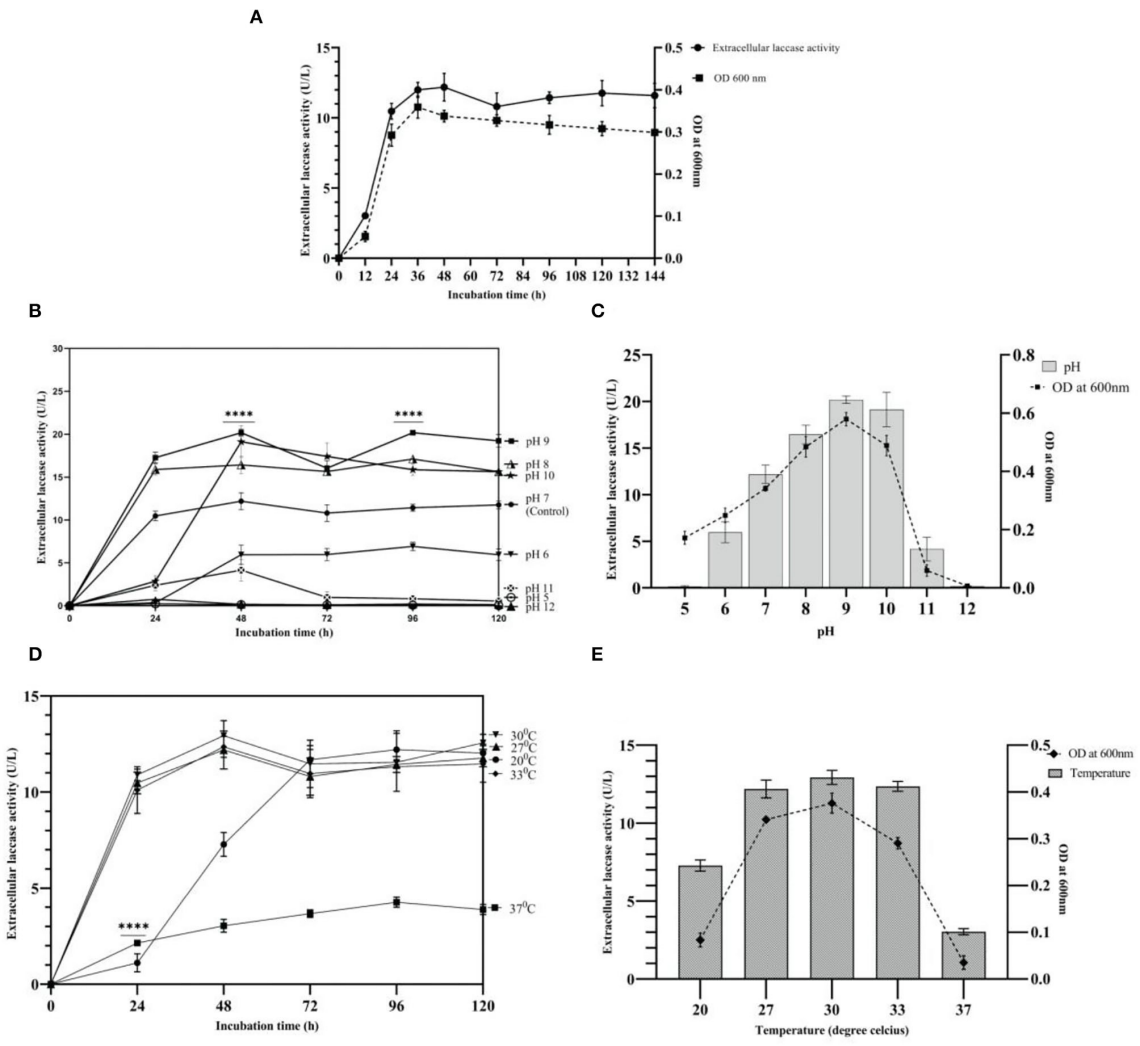
**(A)** Growth and laccase activity profiles of strain AORB19. **(B)** Effect of initial pH values on laccase production; **(C)** Effect of pH on growth and laccase production at 48h; **(D)** Effect of temperature on laccase production; and **(E)** Effect of temperature on growth and laccase production at 48h. The four stars (****) denoted the statistical significance according to the Dunnett's multiple comparison test at P < 0.0001.

Growth and laccase production by strain AORB19 were studied in L-CDB medium up to 144 h. Laccase production in the L-CDB media was easily detectable after 12 h of incubation, increased significantly during the exponential phase (12–36 h), and peaked at the early stationary phase ~ 48 h of incubation. Afterward, the laccase activity showed a slight decrease but more or less maintained stable throughout the rest of the incubation time. The cell growth and density curve based on OD600 readings corresponds well to the enzymatic activity curve (Figure 2A), suggesting that the cell growth stopped and entered the stationary phase at ~48 h.

### Effect of pH and Temperature

The effect of pH on strain AORB19 extracellular laccase activity was explored by adjusting the initial cultural media pH from 5 to 12 and culturing the cells for 120 hours (Figure 2B). Maximum activities were achieved at different pHs, and the higher laccase activities were detected with neutral to weak alkaline media (pH 7–10). To explore whether the impact of media pH on laccase activity was due to the impact on cell growth, the laccase activities and cell densities of 48-h cultures were compared and indeed a higher cell density corresponds well to a higher laccase activity (Figure 2C).

The growth and enzyme yield were both adversely affected by an acidic pH (5.0). Compared with control pH 7 (12.18 U/L), laccase production reached its maximum at pH 9 (*P* < 0.0001, 20.18 U/L) with a 65% increase, followed by a 56% increase (19.13 U/L) at pH 10, and a 34% increase (16.44 U/L) at pH 8. The maximum bacterial biomass at pH 9 indicated the alkaliphilic nature of strain AORB19. However, a higher pH (11) inhibited the cell growth as much as lower pH (5) and at pH 12, cell growth was totally inhibited. Strain AORB19 was cultivated for 120 h at various temperatures ranging from 20 to 37°C to determine the optimal temperature for laccase production. Similarly, the higher laccase activities were detected between temperatures of 27 and 33°C, and the maximum activities were reached at 48-h incubation. Lower temperature (20°C) showed a lower activity at 48 h and caught up at 72 h. However, at higher temperature (37°C), the laccase activities were consistently low over the whole culturing period (Figure 2D).

To explore whether the impact of laccase activities by different temperatures is related to the cell growth, cells were grown for 48 h and the corresponding cell densities and enzyme activities were monitored (Figure 2E). The highest laccase activity was recorded at 30°C (12.93 U/L), followed by a slight reduction at 33°C (12.35 U/L) and 27°C (12.18 U/L). Much lower activities were observed at 20°C and the lowest (*P* < 0.0001) at 37°C (Figure 2E). Correspondingly, a similar pattern of cell densities was observed, suggesting that the enhanced laccase activity was due to the increased cell growth.

### Influence of Nitrogen and Carbon Sources

**Figure 3:**
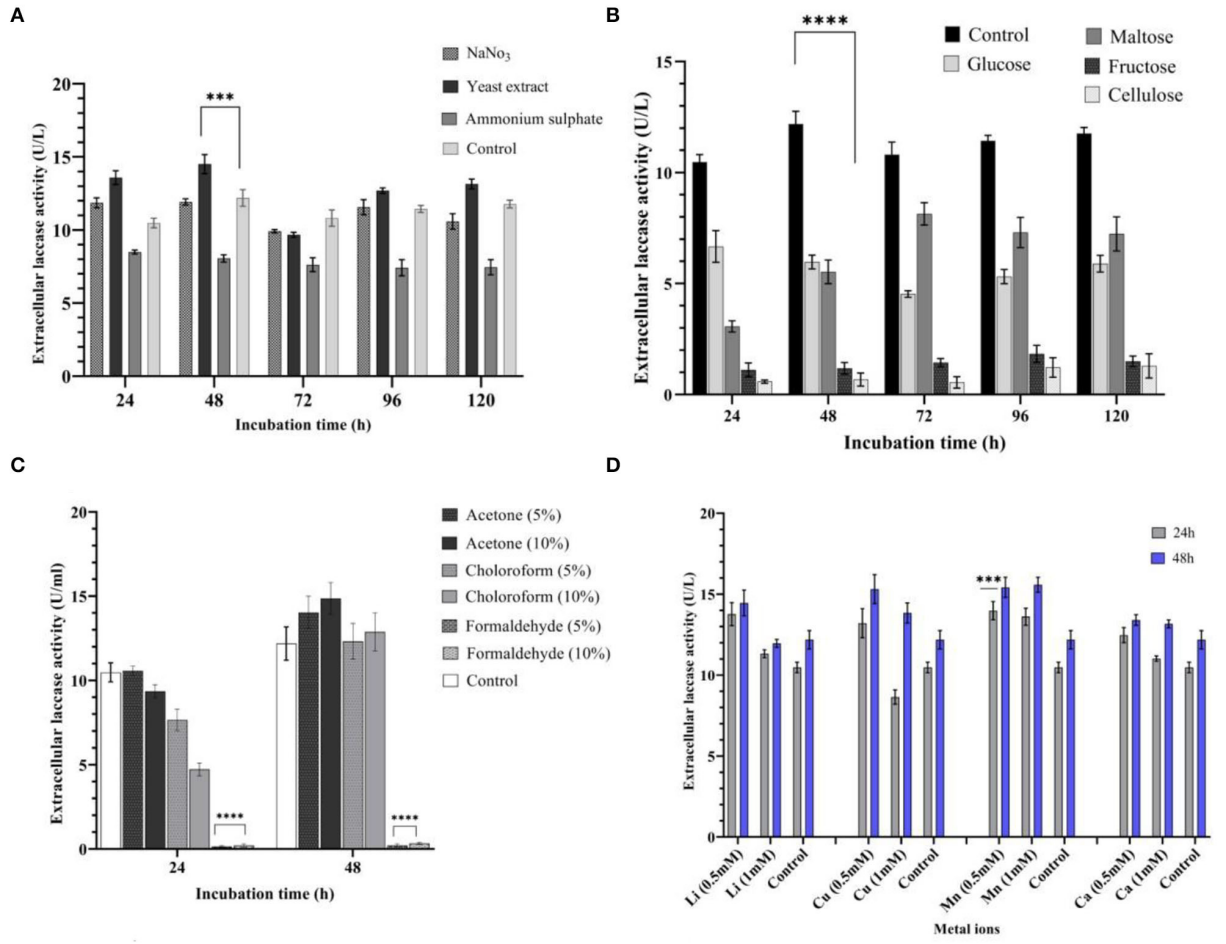
Influence of different media variables on laccase production by strain AORB19. **(A)** nitrogen source; **(B)** carbon source; **(C)** organic solvents; and **(D)** metal ions. The four stars (****) and three stars (***) denoted the statistical significance according to the Dunnett's multiple comparison test at P < 0.0001 and P < 0.001 respectively.

Nitrogen sources, such as ammonium sulfate, yeast extract, and sodium nitrate, were added exogenously to determine their effects on laccase production. An addition of yeast extract to the culture media yielded a 19% increase in laccase production (14.50 U/L) at 48 h, when compared with control medium. When compared with other nitrogen substrates, the yeast extract was shown to be the best nitrogen source in the study (*P* < 0.001), resulting in higher laccase yields (Figure 3A). Ammonium sulfate resulted in a 34% decrease (8.05 U/L) in laccase production at 48 h. Different nitrogen sources were rated in terms of their impact on laccase production: yeast extract > sodium nitrate > ammonium sulfate.

A carbon source of the original medium (sucrose, control) was replaced with fructose, maltose, xylose, and cellulose, each of which was tested individually for impacts on the laccase activities. Among the tested carbon sources, none performed better than the control with the following rank: sucrose (12.18 U/L) > glucose (5.96 U/L) > maltose (5.52 U/L) > fructose (1.18 U/L) > cellulose (0.76 U/L). Particularly, the latter two substitutions significantly lowered (*p* < 0.0001) laccase production (Figure 3B).

### Influence of Metal Ions and Organic Solvents on Laccase Production

The low and high molecular weight organic solvents, such as acetone, formaldehyde, and chloroform, were added to the medium in two concentrations (5 and 10%). It was noted that acetone (5 and 10%) enhanced laccase production by 15 and 21% at 48 h (14.03 and 14.86 U/L, respectively). During the initial phase of incubation (24 h), the presence of chloroform (5 and 10%) lowered laccase production (7.65 and 4.73 U/L, respectively), but this was gradually increased by 48 h. On the other hand, laccase production was significantly inhibited (*p* < 0.0001) when formaldehyde was present in the culture media (Figure 3C).

Cations Mn^2+^, Cu^2+^, Ca^2+^, and Li^+^ (0.5 and 1 mM) were separately supplemented to the basal medium to determine their effects on laccase production by *Serratia proteamaculans*. All metal ions used in the study stimulated laccase yield positively at 0.5 mM concentration. An addition of Mn^2+^ (0.5 and 1 mM) increased laccase production by 33% (*P* < 0.001) and 29% at 24 h (13.98 and 13.60 U/L, respectively) and 25 and 10% at 48 h (15.57 and 15.35 U/L, respectively). Furthermore, the addition of Li^+^ and Ca^2+^ (0.5 mM) improved laccase production by 31 and 18% at 24 h (13.77 and 12.47 U/L, respectively). Cu^2+^ (0.5 mM) enhanced laccase production by 25% at 24 and 48 h (13.20 U/L) consistently. However, at 1 mM concentration, laccase production showed a decline in the presence of Cu^2+^ at 24 h (8.64 U/L), which was followed by an increase of 13% (13.83 U/L) at 48 h (Figure 3D).

### Influence of Media Variables

To reach and hold the points of optimum levels, experiments were performed on culture media by varying one media variable at a time to monitor cell growth and laccase production. Different media variables affecting laccase production were tested under submerged fermentation by inoculating 1% v/v (O.D.600 nm, 0.1) of freshly grown overnight culture of AORB19 strain on 50 ml L-CDB. The culture suspension was withdrawn periodically as per the factor analyzed, and the supernatant was measured for laccase activity with 2,6-DMP assay. A control was also included with all the factors present except the one being studied. Subsequently, submerged fermentation with all the factors at optimum condition was carried out and compared to compute the fold increase in laccase production.

**Figure 4:**
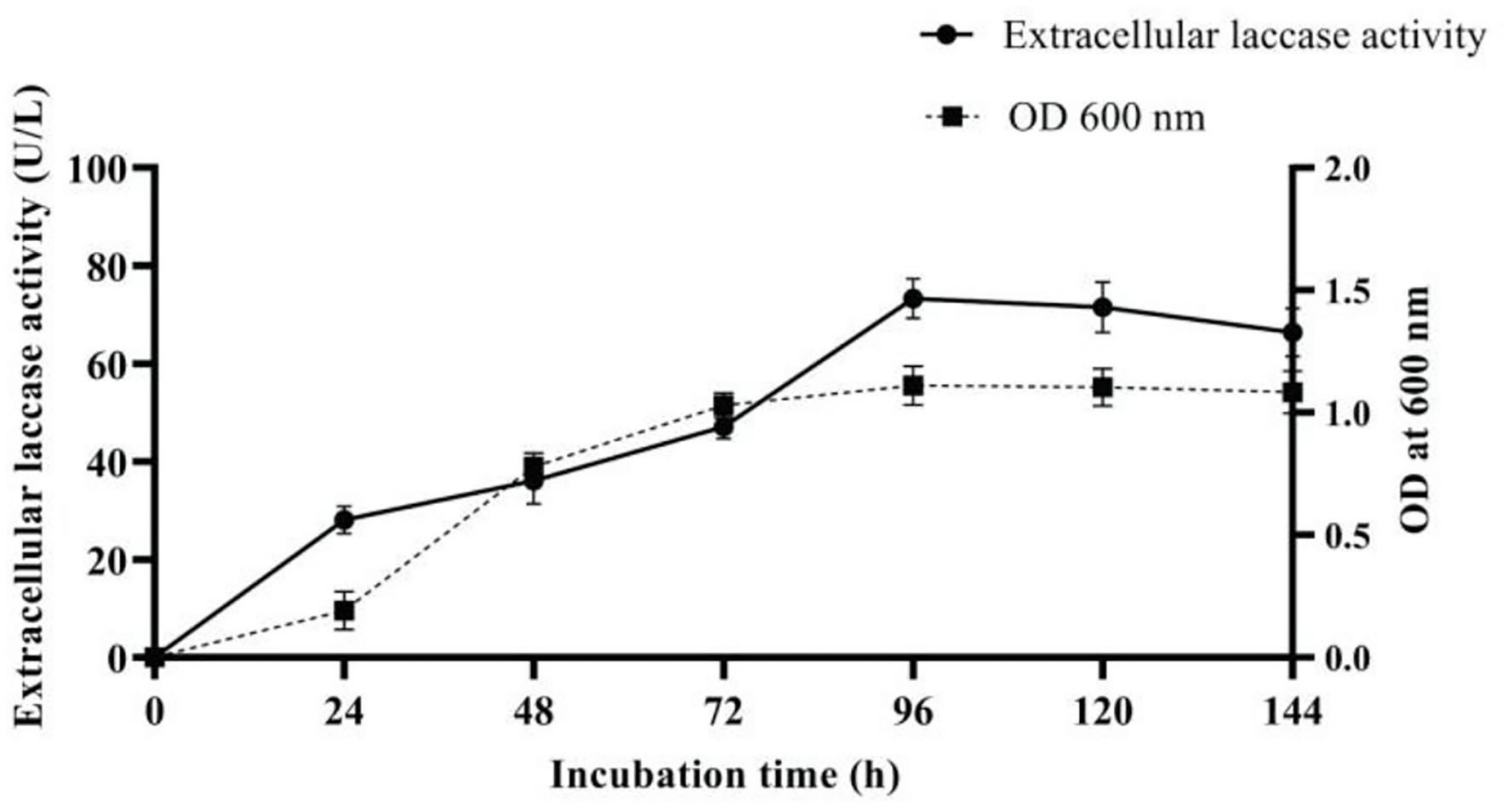
Extracellular laccase production by strain AORB19 using optimized media.

The variables enhancing laccase production were as follows: temperature 30°C, pH 9, yeast extract (2 g/l), Li^+^, Cu^2+^, Ca^2+^, and Mn^2+^ (0.5mM), and acetone (5%). Under optimal condition, a 6-fold increase (73.3 U/L) in laccase production (Figure 4) was achieved when compared with the initial pre-optimized media condition (12.18 U/L).

## Discussion

**Figure 5:**
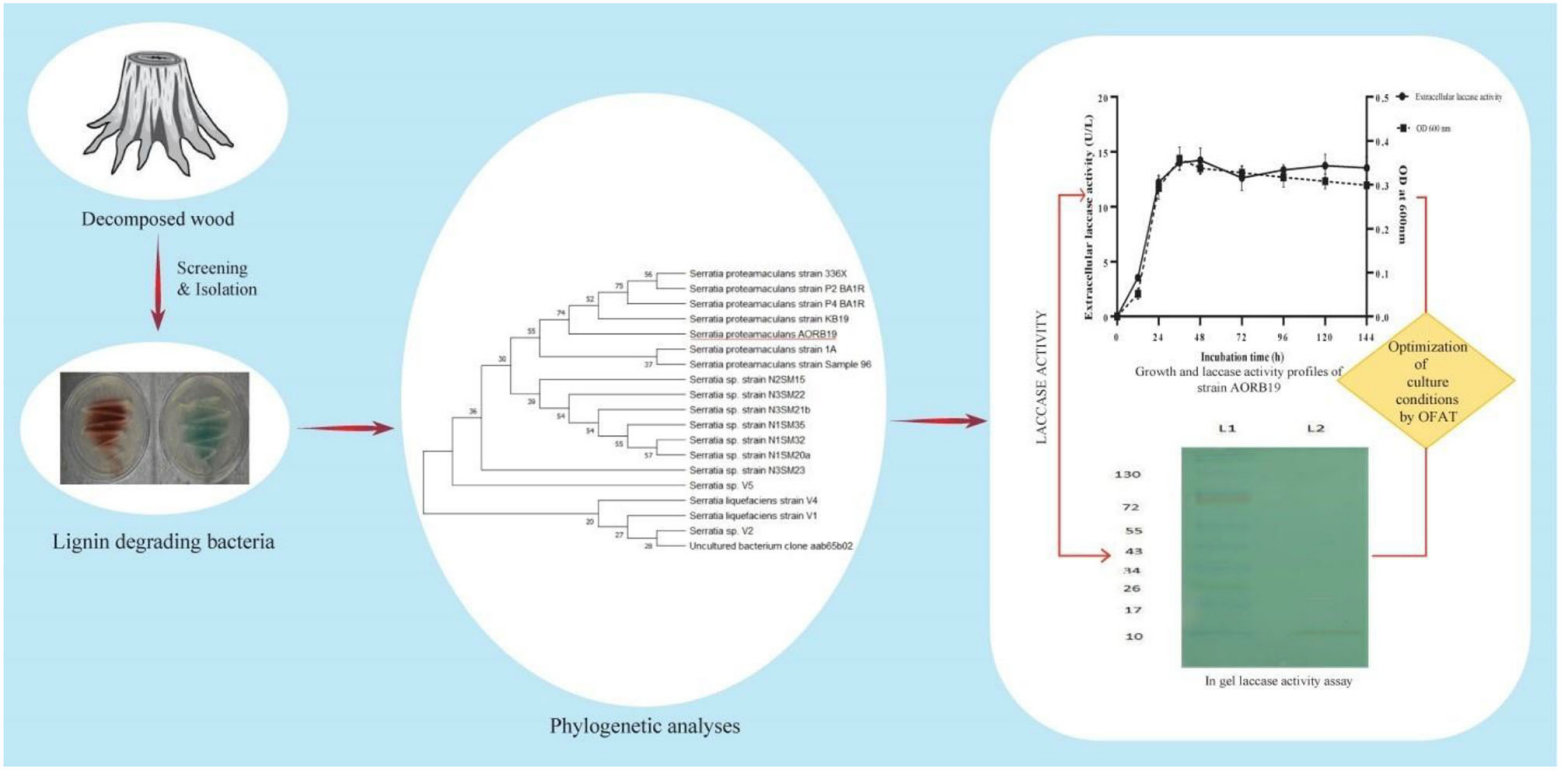
Schematic representation for identification and characterization of laccase producing bacterial strain AORB19.

As the quest for economic, novel, and robust enzymes becomes increasingly important for biotechnological applications, more attention has been paid to systematic exploitation of microorganisms through natural biodiversity screenings and optimization of cultural conditions to produce abundant enzymes that are affordable as well as stable and active under desired operational conditions. By screening microorganisms of decayed wood stumps in this study, we have isolated a bacterial strain that naturally secreted a significant amount of laccase enzyme activities (Figure 5).

The selected strain was characterized phenotypically based on its morphological, physiological, and biochemical characteristics. Unlike many other species in the genus Serratia (Grimont et al., 1977), the identified bacterial strain produced non-pigmented colonies with a characteristic mucoid texture on nutrient agar. The biochemical reactions such as l-arabinose, ornithine and lysine decarboxylase, dulcitol, adonitol, d-arabitol, and d-sorbitol fermentation can aid to diversify different biogroups or species within the genus Serratia (Rafii, 2014). The bacterial strain was identified with a high probability score of 99.9% from the MicroScan version 4.4 x databases as *Serratia liquifaciens* complex which included *S. proteamaculans, S. grimesii*, and *S. liquifaciens*. As species-specific identification of *Serratia liquifaciens* complex was not included in the database, the results were further validated using 16S rRNA gene sequencing. The results of 16S rRNA gene sequencing were comparable with the biochemical characterization, and the species showed 99.9% homology to *Serratia proteamaculans*.

Aromatic compounds formed during the lignin degradation process are lethal to many microorganisms and can negatively affect productivities in the fermentation of lignocellulosic hydrolysates (Henson et al., 2018). However, while investigating the lignin-degrading potential, it was found that the isolated strain AORB19 was able to grow well with lignin as a sole carbon source in the culture media and produced copious laccase. In the study by Wang et al. (2020), it was reported that the cell growth in the lignin-based culture medium is linked to lignin degradation by extracellular enzymes. Thus, *Serratia proteamaculans* AORB19 possesses an important phenotype of lignin-degrading microorganism in terms of bacterial cell proliferation and laccase production in lignin-based media. Beyond this indication, the bacterial strain also exhibited exceptionally stable laccase activity in the culture medium for a prolonged period, suggesting an inherent tolerance of the strain and its enzymes to end-product accumulation during lignin degradation and metabolism.

Microbes produce extracellular enzymes to break down complicated organic materials into usable compounds that can be transported through the cell membrane (Ramin and Allison, 2019). The time-course analysis of strain AORB19 revealed extracellular laccase production throughout the growth phase following a short lag phase. During the exponential phase (12–36h), there was a marked increase in laccase production in the growth medium and peaked at the early stationary phase, reaching the maximum by 48 hours of incubation. The time for optimum laccase production varies depending on the microorganism. For *Pseudomonas extremorientalis* BU118 and *Bacillus subtilis* MTCC 2414, 24 h and 96 h were reported, respectively (Muthukumarasamy et al., 2015; Neifar et al., 2016).

The temperature and pH of the culture medium have a substantial impact on the strain's cell growth and metabolite synthesis. Although the production of laccase was estimated between pH 5 and 12, the optimal range of pH was about 7–10, which corresponded to the optimal cell growth in the study. It was noticed that the laccase production remained steady throughout the incubation period except minor ups and downs during the stationary phase of incubation. Laccase production from other species of Serratia has been reported around a neutral to alkaline pH range and in mesophilic temperature (Chandra et al., 2012). For strain AORB19, the optimal laccase production and cell growth occurred between 27 and 33°C similar to *Bacillus* sp., 30°C (Sondhi and Saini, 2019).

As nitrogen is a fundamental component of proteins and nucleic acids, it is widely known that it is one of the most important nutrients for microbial metabolism (Hernández et al., 2015). For strain AORB19, yeast extract was the most effective nitrogenous source and was consistent with a previous discovery regarding *Bacillus subtilis* DS (Kumar et al., 2018). In this study, maximum laccase production was noticed while using sucrose as the carbon source consistent with a study by Muthukumarasamy et al. (2015) for *Bacillus subtilis* MTCC 2414 strain. Cellulose and fructose significantly inhibited laccase production. While fructose is readily assimilable, cellulose may increase medium viscosity and thus lower oxygen supply and interfere with cell division and metabolic rate (Othman et al., 2018).

Laccase production of strain AORB19 was enhanced by the organic solvents; acetone and chloroform with acetone exhibited a higher impact. Such inductions by organic solvents have also been reported with *Serratia marcescens* and fungus GBPI-CDF-03, and the results were in concurrence with prior studies (Dhakar and Pandey, 2013; Kaira et al., 2015; Wu et al., 2019). Our study showed that several metal cations improved strain AORB19 laccase production. Copper was found to induce laccase production in MTCC2414 (Muthukumarasamy et al., 2015). At 0.5 mM, Cu^2+^ increased laccase production, but at 1 mM, Cu^2+^ initially lowered laccase production at 24 h and then increased enzyme production on further incubation. This reversal can be explained by the considerable toxicity of copper at greater concentrations, as well as the restriction of normal metabolic pathways caused by redox cycling of ions. As a result, laccase production could be viewed as a regulating system used by bacteria to survive the toxicity of inorganic materials like metal ions by changing their oxidation status under aerobic conditions (Kaur et al., 2019).

Laccase synthesis by microorganisms can be influenced by many factors such as chemical and nutritional composition of the media; the optimization approach used may be strain specific. In this study, factors [temperature 30 °C, pH 9, yeast extract (2g/l), Li^+^, Cu^2+^, Ca^2+^, and Mn^2+^(0.5mM), and acetone (5%)] were used to enhance laccase synthesis using a one-factor-at-a-time strategy, resulting in a 6.0-fold overall increase (73.3 U/L) in laccase yield. However, by employing a statistical approach of culture media optimization using five factors by response surface methodology, a 5.5-fold increase in laccase yield (58 U/L) was reported for *Pseudomonas putida* LUA15.1 (Verma et al., 2015). Similarly, *Streptomyces psammoticus* MTCC 7334 produced 3-fold increase in laccase using response surface methods (Niladevi et al., 2009). Furthermore, when compared with the unoptimized media (2.05 U/ml), a 4-fold increase in laccase production in *Bacillus cereus* TSS1 (9.03 U/ ml) was attained after altering the media variables using response surface approach (Rajeswari et al., 2015).

## Conclusion

Taken together, a new bacterial strain *Serratia proteamaculans* AORB19 has been characterized that stably secreted high-level laccase activity after 24-h culture over an extended culture time. The combination of selected culture conditions resulted in a 6.0-fold increase in laccase production without the addition of any toxic inducers. This is, to our knowledge, the first report on the natural production and parameter selection for the expression of extracellular laccase by a *Serratia proteamaculans* strain. The isolated bacterial strain preferred weak alkaline pH and mesophilic culture conditions that are desired traits for many industrial applications. This first phase of research focused on identification and characterization of a new bacterial strain and screening of important factors that influence laccase production using the OFAT strategy. Future experiments will be designed to explore the impact of other important factors such as the amount of oxygen in the culture medium on enzyme activity and the interactions among different nutritional and conditional parameters and to identify the optimized conditions by the statistical method—Design of Experiment and Response Surface Methodology. The final optimized process should allow secretion of further increased amount of laccase that confers strain AORB19 greater potential to be either directly used in fermentation process or for laccase enzyme production.

In addition, the high-level enzyme expression would expedite the biochemical purification and characterization of the specific AORB19 laccase. A greater understanding of the functionality of this bacterial enzyme will likely lead to its practical application in lignin valorization and other biotechnologies that focus on cost-effective biomass conversion and environmental remediation.

## Data Availability Statement

The original contributions presented in the study are included in the article/Supplementary Material, further inquiries can be directed to the corresponding authors.

## Author Contributions

NA carried out the majority of experiments and drafted the manuscript. FH set up laccase assay conditions and assisted in experiments. TY and WQ conceived the concept, designed the experiments, reviewed the manuscript, and coordinated the entire study. The final version of the manuscript was read and approved by all authors.

## Funding

This manuscript represents the National Research Council Communication # 58306. The research of this project was funded by the NRC New Beginning Fund of the National Program Office Ideation Programs and the NRC Industrial Biotechnology Program to TY. NA was supported by the graduate scholarship of Lakehead University.

## Conflict of Interest

The authors declare that the research was conducted in the absence of any commercial or financial relationships that could be construed as a potential conflict of interest.

## Publisher's Note

All claims expressed in this article are solely those of the authors and do not necessarily represent those of their affiliated organizations, or those of the publisher, the editors and the reviewers. Any product that may be evaluated in this article, or claim that may be made by its manufacturer, is not guaranteed or endorsed by the publisher.
