# Supplementary material for: Identification and Characterization of a New Serratia proteamaculans Strain That Naturally Produces Significant Amount of Extracellular Laccase
Source: Front Microbiol. 2022 Jul 18;13:878360. doi: 10.3389/fmicb.2022.878360 (PMC9339997; doi:10.3389/fmicb.2022.878360)

Supplementary Figure 1 Agarose gel electrophoresis of the amplified 16SrRNA gene of bacterial strainAORB19. (Lane 1: 1 Kb DNA ladder, Lane 2: amplified DNA of strain AORB19).

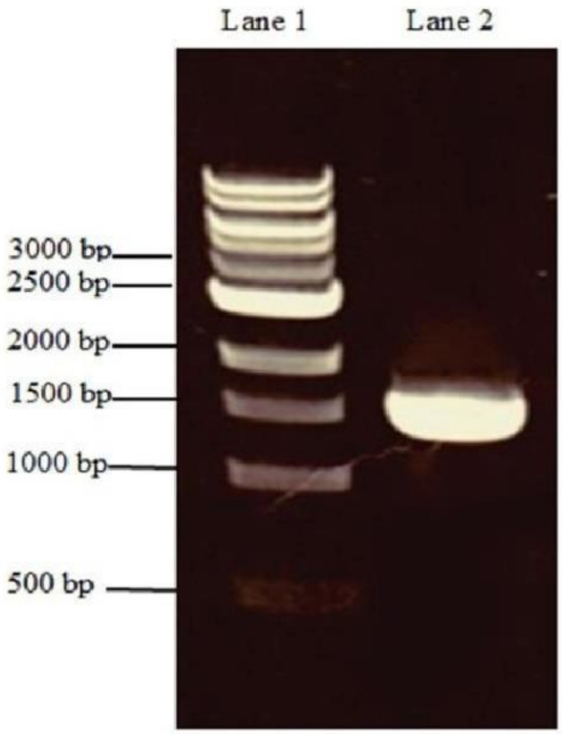

Supplement: Supplementary file 1 [file Data_Sheet_1.PDF]
