# Supplementary material for: Identification and Characterization of a New Serratia proteamaculans Strain That Naturally Produces Significant Amount of Extracellular Laccase
Source: Front Microbiol. 2022 Jul 18;13:878360. doi: 10.3389/fmicb.2022.878360 (PMC9339997; doi:10.3389/fmicb.2022.878360)

Supplementary Figure 2 Phylogenetic tree of strain AORB19 showing the relationship with 18nucleotide sequences of closely related neighboring species**.**

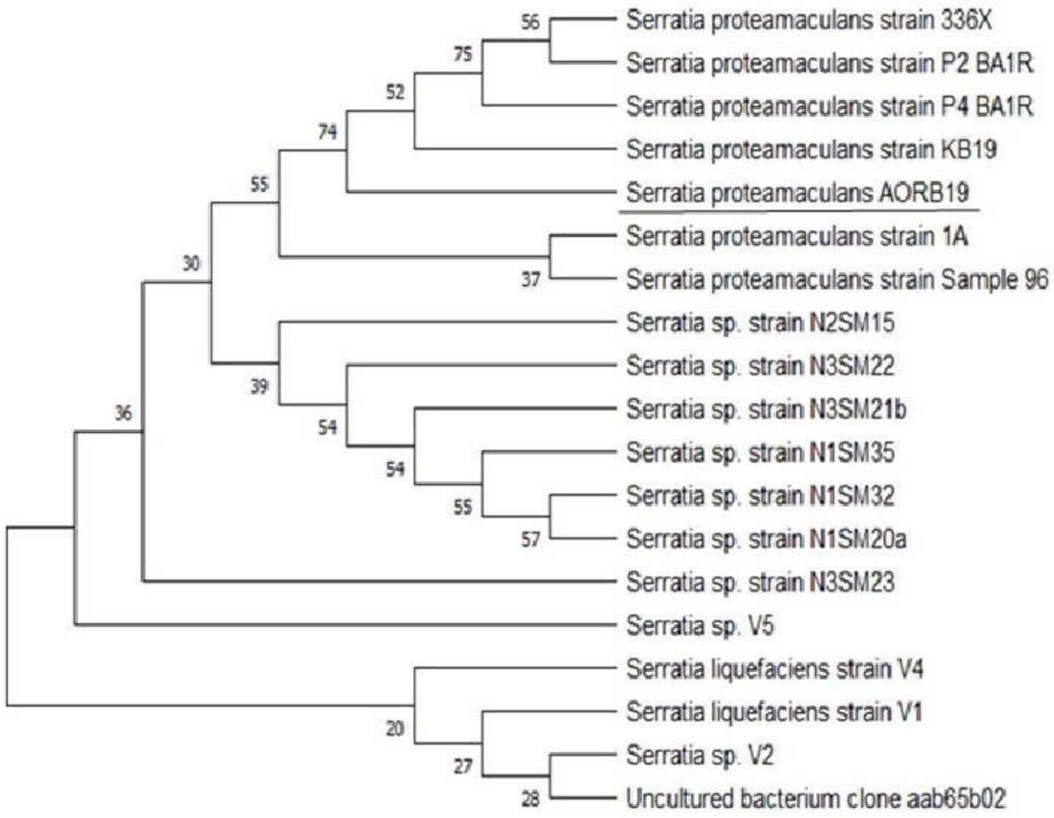

Supplement: Supplementary file 2 [file Data_Sheet_2.PDF]
