# Supplementary material for: Identification and Characterization of a New Serratia proteamaculans Strain That Naturally Produces Significant Amount of Extracellular Laccase
Source: Front Microbiol. 2022 Jul 18;13:878360. doi: 10.3389/fmicb.2022.878360 (PMC9339997; doi:10.3389/fmicb.2022.878360)
Supplement: Supplementary file 3 [file Data_Sheet_3.PDF]

Supplementary Table 1 Biochemical characterisation of AORB19 in gram negative ID Type 2 (NID2) panel

Characteristic features	AORB19
**Carbohydrate fermentation**	
Glucose	+
Sucrose	+
Inositol	+
Raffinose	+
Adonitol	-
Rhamnose	-
Arabinose	+
Sorbitol	-
Melibiose	-
**Substrate utilization**	
Oxidase	-
Cetrimide	-
Arginine	-
Lysine	+
Ornithine	+
Tryptophan deaminase	-
Esculin	-
o-Nitrophenyl-D-Galactopyranoside	+
Citrate	+
Malonate	-
Acetamide	-
Tartrate	-
Hydrogen sulfide	-
Indole	-
Nitrate	+
Voges-proskauer	+
Urea	-
**Growth in the presence of antibiotics**	
Penicillin G 4 g/mL	-
Kanamycin 4 g/mL	-
Colistin 4 g/mL	-
Cephalothin 8 g/mL	+
Nitrofurantoin 64 g/mL	-
Tobramycin 4 g/mL	-
